# Effect of Anaerobic Treatment on the Formation of Volatile Flavor Characteristics in GABA White Tea

**DOI:** 10.3390/foods14071153

**Published:** 2025-03-26

**Authors:** Dan Su, Zhixia Wang, Jia Zhou, Hongtao Ren, Mufang Sun, Hongjie Zhou

**Affiliations:** 1 College of Tea, Xinyang Agriculture and Forestry University, Xinyang 464000, China; 2022360002@xyafu.edu.cn; 2 Dabie Mountain Laboratory, Xinyang 464000, China; 3 Key Laboratory for Evaluation and Utilization of Gene Resources of Horticultural Crops, Ministry of Agriculture and Rural Affairs of China, Hunan Agricultural University, Changsha 410128, China; wangsophia12@163.com; 4 College of Tea, Yunnan Agricultural University, Kunming 650000, China2004129@ynau.edu.cn (H.R.)

**Keywords:** GABA white tea, volatile compounds, freshness and fragrance, Yunnan white tea

## Abstract

This study investigated the volatile flavor characteristics of Fujian white tea (FWT), Yunnan white tea (YWT), and GABA-enriched white tea (GWT) using headspace solid-phase microextraction combined with gas chromatography-mass spectrometry (HS-SPME-GC-MS). Cluster analysis and sensory evaluations were employed to compare the relative content changes in volatile compounds and their contributions to freshness-related aroma. A total of 85 volatile compounds were identified, with cis-3-Hexenyl isovalerate, *β*-Ocimene, and nerol identified as key contributors to the fresh aroma of white tea. Comparative analysis of 2023 and 2024 GABA white tea batches revealed significant differences in volatile compounds, highlighting the role of anaerobic treatment in enhancing freshness and purity. The findings provide theoretical support for optimizing GABA white tea production and advancing functional tea research.

## 1. Introduction

White tea, primarily produced in Fuding and also cultivated in other regions, such as Zhenghe and Jianyang within Fujian Province, is one of the six traditional types of tea in China. Its production methods have evolved over centuries, taking on a more recognizable form during the late Qing Dynasty. White tea has a fresh and refreshing taste, characterized by a unique pekoe aroma and floral, fruity notes, complemented by a subtle scent of young buds. Furthermore, white tea is cherished by consumers not only for its unique flavor profile but also for its health benefits, including free-radical scavenging activity [1], antioxidant activity [2], and neuroprotective effects [3]. Based on quality and processing methods, white tea can be categorized into various grades, such as Baihaoyinzhen, Baimudan, Gongmei, and Shoumei, with Baimudan and Shoumei being particularly popular among consumers due to their accessibility and affordability [4].

As the least processed category of tea, white tea is generally produced by simply drying fresh tea leaves [5]. The drying process plays a pivotal role in shaping white tea’s aroma profile by driving biochemical transformations that increase aroma-active components [6]. Drying plays a crucial role in shaping the characteristic floral aroma of white tea. In fact, during the drying stage, the biochemical changes that occur within the fresh leaves contribute to the transformation of the contents, especially polyphenols, which are important for reducing the astringency of the tea broth and increasing the sweetness of the tea broth. In addition, a variety of compounds in tea undergo complex chemical reactions, such as the Maillard reaction and Strecker degradation, which result in the production or increased concentration of a range of aromatic substances, including esters, ketones, heterocyclic compounds, and hydrocarbons. It is worth noting that further volatilization of low-boiling-point aromatic substances reduces the grassiness, while aroma components such as the miller’s scent and floral aroma are enhanced, resulting in a more complex and fascinating aroma of the final product.

The withering stage is also an important process in the production of white tea aroma [7], Long-term withering is a key step in the formation of the aroma of white tea by regulating the characteristic aroma of volatile compounds [6]; therefore, the aroma of white tea is highly correlated with the processing. Glucoside and carotenoid degradation products such as geraniol, linalool, and ionone contribute to the floral and sweet characteristics of white tea [7], while Strecker degradation products such as phenylethylaldehyde contribute to the fruity and fresh character. Linalool and methyl salicylate are important volatiles that contribute to aroma formation, and both complete the production and interconversion of volatiles at the processing stage. At present, research on white tea mainly focuses on the differences in the aroma components of different grades, as well as the characteristics and key aromas of *γ*-aminobutyric acid (GABA) white tea [8], while there are few studies on the development of functional white tea.

GABA is an amino acid that exists widely in vegetables, fruits, and fermented foods. It can inhibit a rise in blood pressure, fight epilepsy [9,10], prevent depression, and fight oxidation [11], and at the same time is one of the important amino acids in the human body. *γ*-aminobutyric acid is an inhibitory neurotransmitter in the central nervous system and has a variety of physiological functions [12]. It is difficult to be absorbed only from the daily diet, hence, it is necessary to develop *γ*-aminobutyric acid-related products that are easily absorbed by the human body. GABA white tea is a functional variant made from the fresh leaves of Yunnan tea plants through a process involving spreading the leaves and then anaerobic fermentation taking place for six hours [13]. In terms of the content of GABA tea, by studying the physical and chemical components of GABA tea and green tea, it was found that the main difference between GABA tea and green tea lies in the content of various amino acids [10]. However, there are a lack of studies comparing the volatile compound compositions between GABA tea and traditional tea. Research on GABA tea has mainly focused on its efficacy, but little has been performed on the flavor and taste of GABA tea. In this study, five kinds of tea samples of Fuding white tea, Yunnan white tea, and GABA white tea were used as research objects, and the differences in volatile substance composition of the five kinds of white tea were analyzed by using headspace solid-phase microextraction combined with gas chromatography-mass spectrometry (HS-SPME-GC-MS) [14]. The aroma characteristics of GABA white tea were explored to provide a theoretical basis for the development of functional white tea. This study is the first to systematically compare the differences in volatile compositions between GABA white tea and traditional white tea to supplement the theory of functional white tea flavor research.

## 2. Materials and Methods

### 2.1. Materials and Reagents

Materials: Five samples were selected for this study, which were collected from Fuding City in Fujian Province and Jinggu County in Yunnan Province during 2023 and 2024, respectively. The varieties included Fuding big-white tea from Fuding and Jinggu big-white tea from Yunnan. Fuding white tea was produced using the traditional method and harvested in 2023 (designated as FWT) and 2024 (FWT1) (Daqin Tea Industry Co., Ltd., Fuzhou, China). Yunnan white tea also had two treatments: one followed the traditional process and was harvested in 2023 (YWT); the other included an additional anaerobic step, with samples taken in 2023 (GWT) and 2024 (GWT1) (Dalishu Co., Ltd., Dali, China). To ensure quality, sensory evaluations were performed on all three samples following the national standard GB/T 23776-2018 [15] for tea sensory assessment, which helps capture the aroma profiles of the different white tea varieties. Prior to analysis, the tea samples were prepared in accordance with the national standards GB/T 8302-2013 [16] for tea sampling and GB/T 8303-2013 [17] for grinding, sample preparation, and dry matter content determination, and then stored at −4 °C until use.

Reagents: All reagents used in this study had an analytical purity exceeding 98%. Ethyl decanoate (Sigma-Aldrich LLC, Shanghai, China. internal standard) was used. An internal standard (ethyl decanoate) is added to a head empty bottle for the semi-quantification of volatile compounds. C7−C40 n-alkanes (TCI, Shanghai, China) were used to determine linear retention indices (LRIs).

### 2.2. Methods

#### 2.2.1. Extraction of Volatile Compounds

The headspace solid-phase microextraction (HS-SPME) parameters were as follows: 1.0 g of tea powder was first placed into a 20 mL sealed glass vial and 5 mL of boiling deionized water was added, then 1.00 µg of ethyl caprate was added as internal standard and the bottle sealed. For the CTC autosampler, the vial was immediately put into a thermostatic oscillator and kept at 60 °C. After 10 min of stabilization, the volatiles were adsorbed for 50 min by using a carboxy/polydimethylsiloxane (CAR/PDMS) coating fiber (Supelco, Inc, Bellefonte, PA, USA), with a rotating speed of 250 r/min. Finally, the volatiles were desorbed at 250 °C for 5 min in the GC×GC-TOFMS injector.

#### 2.2.2. Analysis of Volatile Substances by GC-MS

Identification of the volatile compounds in the tea aroma extract was performed by a Shimadzu gas chromatograph 2010-plus, with a triple quadrupole mass spectrometer QP 2020 (Shimadzu, Shanghai, China). GC conditions were improved based on previous studies [18,19]. The employed GC column was an SH-Rxi-5Sil MS capillary column (30 m × 0.25 mm × 0.25 μm). The temperature of the injection port was set at 250 °C in splitless mode. Helium (purity > 99.999%) was applied as the carrier gas. The initial temperature was 50 °C for 5 min, and the heating rate was 6 °C/min to 250 °C for 15 min. The split ratio was set not to split, the mass spectrometry conditional ion source was EI, the temperament interface temperature was 280 °C, the ion source temperature was 230 °C and the four-bar temperature was 150 °C.

#### 2.2.3. Analysis of Volatile Components

For qualitative analysis, we referenced the National Institute of Standards and Technology (NIST) standard library to match (≥80) the detected substances. Then, we conducted the final qualitative analysis according to the relative retention time of each volatile substance.

For quantification analysis, the internal standard method was used to quantify the volatile flavor compounds in the aroma of tea. Ethyl caprate was selected as the internal standard (10 mg/kg tea sample). The calculation formula is as follows:

$$f=\frac{\frac{A_{s}}{M_{s}}}{\frac{A_{r}}{M_{r}}}$$

where *A_s_* and *A_r_* are the peak areas or peak heights of the internal standard and control, respectively, and *M_s_* and *M_r_* are the amounts of the internal standard and control added, respectively. Then, a sample of the component solution containing the internal standard was taken, the chromatogram recorded, and the content (*M_i_*) calculated from the peak response of the component solution containing the internal standard. This was done using the following formula:

$$M_{i}=\frac{f\times A_{i}}{\frac{A_{s}}{M_{s}}}$$

where *M_i_* is the analyte concentration in mg/kg, *A_i_* and *A_s_* are the peak area or peak height of the substance to be measured and the internal standard, respectively, and *M_s_* is the amount of internal standard added.

#### 2.2.4. GABA White Tea Anaerobic Treatment

The production process of GABA white tea is as follows: fresh leaf plucking, anaerobic treatment, withering, and drying. Among them, the fresh leaves were selected from Yunnan Province’s Jinggu big-white tea variety, and the picking standard is one bud and two or three leaves. The harvested fresh leaves were loaded into a special fermenter for vacuum anaerobic nitrogenation (N_2_ ≥ 98%) for 6 h. The experimental parameters were vacuum degree of 0.04 mpa, nitrogen filling pressure of 0.006 mpa, and it was set in three parallel groups [20].

#### 2.2.5. Sensory Evaluation

Five trained group members (three men and two women) from Xinyang Agriculture and Forestry University were selected to conduct sensory evaluations of tea samples. Prior to the formal experiments, each assessor underwent at least 90 h of specialized training to ensure they could accurately describe the sensory characteristics of tea. During the evaluation, the assessor is required to evaluate the appearance, soup color, aroma, taste, and leaf base of each sample and provide the key terms to describe quality characteristics collectively according to the Methodology for Sensory evaluation of Tea [15]. A total of 3 g of sample was placed in a special cylindrical evaluation cup, added with 150 mL boiling water, covered, soaked for 5 min, and strained into the evaluation bowl to evaluate. Each assessor was evaluated independently without any discussion. Each sample was repeated three times. After each evaluation, the next sample was evaluated after 10 min at rest. The evaluation environment requirements were that it was clean and odorless, with a temperature of 20–25 °C. The average value of the evaluation results of the five evaluators was taken as the final result of the sample.

#### 2.2.6. Data Analysis and Statistics

All the above analyses were performed in 3 replicates for each sample and the average values are presented. A one-way ANOVA based on replicate data was carried out using the SPSS 25package and cluster analysis was carried out by MetaboAnalyst 6.0 (https://www.metaboanalyst.ca/, accessed on 3 January 2025). Venn diagrams and bar charts were made using Origin 2021.

## 3. Results and Discussion

### 3.1. Identification of Volatile Components of Three Types of White Tea

The volatile organic compounds (VOCs) in the three types of white tea varieties were systematically analyzed using headspace solid-phase microextraction coupled with gas chromatography-mass spectrometry (HS-SPME-GC-MS). A total of 85 distinct volatile compounds were identified and categorized as follows: 12 alkenes; 10 ketones; 7 aldehydes; 9 aromatic substances; 7 alcohols; 4 heterocyclic compounds; 23 alkanes; 10 esters; and 3 phenols (Table 1). The most abundant variety in terms of volatile compounds is FWT with 64 different compounds, followed by GWT with 55 compounds and YWT with 49 compounds.

### 3.2. Analysis of Volatile Substances in Three Types of White Tea

**Figure 1:**
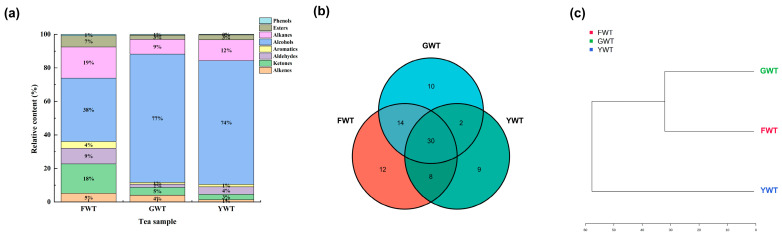
Distribution of the relative content of volatiles of the three types of white teas. (**a**): Type and percentage of volatile substances. (**b**): Venn diagram. FWT: Fuding white tea; GWT: GABA white tea; YWT: Yunnan white tea. (**c**): Hierarchical clustering dendrogram.

Based on the relative content of each compound, the percentage of each substance in the different tea samples was calculated. As shown in Figure (Figure 1a), the volatiles of FWT were mainly alcohols (37.76%), alkanes (18.65%), ketones (17.59%), and aldehydes (9.32%); the volatiles of GWT were mainly alcohols (76.55%), alkanes (8.80%), ketones (4.71%), and alkenes (4.03%); while the volatiles of YWT were mainly alcohols (73.70%), alkanes (12.50%), and aldehydes (4.45%). The main volatile components of white tea were proved to be dominated by alcohols, which was consistent with previous studies [6]. The aroma of tea samples is mainly caused by the differential components, with up to 12 unique components in FWT, 10 in GWT, and 9 in YWT (Figure 1b). These unique aroma components not only give various types of tea unique flavor characteristics, but are also one of the important factors for distinguishing between different varieties of tea [21].

The unique substances in FWT are 2-Bornene (0.81%), 1-(4-tert-Butylphenyl)propan-2-one (0.45%), Isoshyobunone (0.62%), 3-Undecanone(1.30%), 1-ethyl-2,3-dimethyl-Benzene (0.15%), 2-methyl-Naphthalene(0.29%), 1,2-dihydro-1,1,6-trimethyl-Naphthalene (0.25%), *α*-Ionene (0.16%), Phenylethyl alcohol (0.49%), Cyclopentadecane (0.64%), and Diethyl Phthalate(0.53%). 2-Bornene is considered to be one of the main substances responsible for the freshness of white tea [22]; 1,2-dihydro-1,1,6-trimethyl-Naphthalene (OAV:8.2) presents floral, fruity, sweet [23] notes which are probably one of the main contributions of the aroma; *α*-Ionene (OAV:6.38) has a floral and fruity aroma; Phenylethyl alcohol with the floral odor of sweet roses [8,24,25,26]. GWT has a unique composition of trans-*β*-Ocimene (0.23%), *β*-Ocimene (0.87%), (Z)-3-Tetradecene (0.05%), Decanal (0.67%), Indolizine (0.14%), *α*-Terpineol (0.93%), 2,6,10-trimethyl-Dodecane (0.16%), Phthalic acid, isobutyloctyleste (0.22%), and 2-Methyl-4-hydroxyaniline (0.15%). Trans-*β*-Ocimene is floral, rose, and cherry-like [26]; *β*-Ocimene is grassy and floral with hints of orange blossom oil, citrus, and is herbal, spicy, sweet [23]; Decanal has a sweet, citrusy, waxy, floral, orange, and fat taste [27]; *α*-Terpineol has a clove scent, and is sweet [25]. The distinctive volatile compounds in GWT and FWT are floral, fruity, and sweet, but sensory reviews show fresh aromatic qualities. However, it is worth noting the presence of cis-Geranylacetone in both GWT and FWT, which is considered to be one of the main substances in the fresh aroma [22], and thus it can be seen that the main aroma plays a decisive role in the aroma of tea leaves. The only volatile substances available in YWT are 3-carene (0.21%), 2-Butyl-2-octenal (0.10%), (E)-1,2,3-trimethyl-4-propenyl-Naphthalene (0.11%), Isoborneol (0.27%), nonyl-Cyclopentane (0.29%), 2,6,11,15-tetramethyl-Hexadecane (0.12%), undecyl-Cyclopentane (0.12%), Methyl salicylate (0.50%), and 2,4-Di-tert-butylphenol (0.34%). The aroma of Isoborneol is characterized by a near camphor-like odor, whilst Methyl salicylate is minty, wintergreen-like, and fruity [23,25], consistent with the sensory review (Table 2); however, the volatile substances associated with milli incense deserve to be explored in future studies.

2-Bornene, cis-3-Hexenyl isovalerate, and *β*-Ocimene are key substances for the fresh aroma quality of white tea. Since GABA white tea has a stronger effect, the investigation of the aroma quality characteristics of GABA white tea is one of the focuses of this study.

### 3.3. Hierarchical Clustering Analysis of Three Types of White Tea

Hierarchical clustering is an algorithm used in clustering analysis that forms clusters based on the similarity between data points of different categories. Initially, each object is treated as a separate cluster, and the two closest clusters are merged iteratively until all objects belong to a single cluster. Hierarchical clustering can address some limitations of k-means clustering, such as not requiring the pre-determination of the number of clusters (K-value).

Based on the results of hierarchical clustering (Figure 1c), FWT and GWT exhibit higher similarity compared to YWT. Both GWT and YWT use Yunnan big-leaf tea trees as raw materials; however, the production process of GWT includes a vacuum anaerobic step, distinguishing it from the traditional white tea production process of YWT. This additional step influences the oxidation level of GWT during processing, potentially enhancing its quality characteristics.

### 3.4. Anaerobic Treatment Efected the Aroma Characteristics of GABA White Tea

To better investigate the aroma characteristics of GABA white tea, we compared the volatile compounds in GABA white tea samples from 2023 and 2024, identifying a total of 76 volatile compounds using HS-SPME-GC-MS (Table 3). These two types of GABA white tea share 34 volatile compounds, primarily consisting of alcohols, esters, ketones, and alkenes. This profile is similar to that of ordinary white tea, although GABA white tea contains additional volatile compounds that impart a fresh and crisp aroma (Table 4).

For instance, cis-3-Hexenyl isovalerate and nerol both contributed to a notably fresher aroma in the 2024 tea samples. As Table 4 illustrates, the volatile components in GABA white tea can be categorized into four primary aroma profiles: floral and fruity (A), fresh (B), woody (C), and others (D). According to sensory evaluation results, GABA white tea exhibits a fresh and pure aroma, attributed to key compounds such as cis-Geranylacetone, *β*-Ocimene, and nerol. These substances play a decisive role in defining the characteristic aroma of GABA white tea.

### 3.5. Discussions

**Figure 2:**
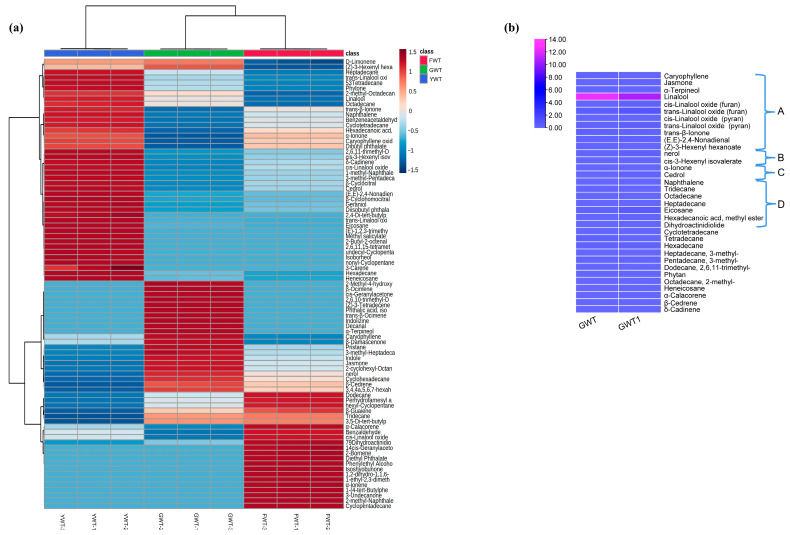
(**a**) Cluster heat map of the relative content of the three white tea species. (**b**) Classification of key volatile substances. A: fruity; B: fresh; C: woody; D: other.

Fuding white tea has a unique flavor and is a typical representative of traditional white tea. Yunnan white tea is an emerging force in the white tea market. Innovative white tea processing technology is of great significance to expand the white tea market. Anaerobic treatment can not only enrich the functions of white tea but also promote the diversification of white tea flavors. Taking Fuding white tea and Yunnan white tea as comparisons, it is helpful to clarify the effect of anaerobic treatment on the formation of aroma quality of GABA white tea. Studies have shown that under enzymatic hydrolysis or humid and hot conditions, catechins in white tea will naturally oxidize, reducing the bitterness of tea soup, and thus improving the quality of tea [29] (Figure 2a). Compared to YWT, GWT was found to contain a total of 32 substances (Figure 2b), with five volatile compounds being upregulated following anaerobic treatment. These include D-Limonene (6.00%), with a lemony aroma; Caryophyllene (96.94%), featuring a pale lilac-like scent; *β*-Damascene (73.12%), known for its pleasant fruity notes; (Z)-3-Hexenyl hexanoate (22.17%), imparting a distinctively fruity green aroma reminiscent of pears; and Dihydroactinidiolide (69.94%). After anaerobic treatment, alkenes have always been considered the main source of tea aroma and ketones constitute one of the primary aroma categories in white tea, while esters are thought to moderate the overall aroma profile. The addition of these substances better modulates the aroma of the tea leaves, giving a more pronounced floral-fruity aroma.

After anaerobic treatment, linalool (47.92%) and Eicosane (88.99%) were significantly downregulated. Some studies have shown that linalool, geraniol, and *β*-ionone are the main substances affecting the aroma characteristics of white tea with different treatments [7,8,21], especially the withering process. Withering under sunlight is conducive to the enrichment of linalool. Linalool is found in a wide variety of teas [28], and its relative content was reduced after anaerobic treatment, possibly due to conversion into other aromatic substances or evaporation during processing. However, due to limited research on how alkanes affect tea leaf aroma, their mechanism of action remains unclear [28]. This represents another promising area for future investigation.

## 4. Conclusions

The determination of volatile substances in Fujian and Yunnan white teas using HS-SPME-GC-MS showed that the aroma components of GABA white tea were richer than those of ordinary white tea. The relative content of volatile substances in GABA white tea was 2.17 times higher than those of different varieties of white tea with small leaves. The relative content of volatile substances in GABA white tea are mainly alcohols. The overall aroma of GABA white tea is characterized by freshness and crispness. Cis-3-hexenyl isovalerate, *β*-Ocimene, and nerol are key to the freshness of the aroma. As anaerobic treatment of GABA white tea can retain more volatiles from the fresh leaves and reduce dissipation during processing, it significantly enhances the milled and clear aroma of GABA white tea. This study provides a theoretical basis for the future development of GABA functional teas.

## Figures and Tables

**Table 1:** Qualitative quantification of volatile substances in three types of white tea.

No.	Compound	RI	RT	CAS	Relative Content		
					FWT	GWT	YWT
Alkenes							
1	D-Limonene	1030.78	11.43	005989-27-5	0.05	0.15	0.15
2	trans-*β*-Ocimene	1041.65	11.75	003779-61-1	/	0.04	/
3	*β*-Ocimene	1051.95	12.06	013877-91-3	/	0.15	/
4	Caryophyllene oxide	1592.80	24.76	001139-30-6	0.05	/	0.07
5	Caryophyllene	1425.55	21.34	000087-44-5	0.05	0.17	0.09
6	*α*-Calacorene	1550.15	23.91	021391-99-1	0.08	0.04	0.05
7	*β*-Cedrene	1649.84	25.84	000546-28-1	0.02	0.03	/
8	*δ*-Cadinene	1529.41	23.50	000483-76-1	0.06	0.05	0.09
9	*β*-Guaiene	1664.45	26.11	000088-84-6	0.04	0.03	/
10	3-Carene	1234.62	16.97	013466-78-9	/	/	0.08
11	2-Bornene	1231.97	16.91	000464-17-5	0.07	/	/
12	(Z)-3-Tetradecene	2215.22	20.63	041446-67-7	/	0.05	/
Ketones							
13	1-(4-tert-Butylphenyl)propan-2-one	1438.03	21.61	081561-77-5	0.04	/	/
14	cis-Geranylacetone	1456.51	22.00	003879-26-3	0.16	/	/
15	Isoshyobunone	1471.18	22.31	1000360-30-1	0.05	/	/
16	Jasmone	1409.54	21.01	000488-10-8	0.06	0.13	/
17	*α*-Ionone	1433.71	21.52	000127-41-3	0.12	0.07	0.13
18	trans-*β*-Ionone	1491.03	22.72	000079-77-6	0.69	0.36	1.04
19	3-Undecanone	1290.73	18.33	002216-87-7	0.11	/	/
20	*β*-Damascenone	2210.65	20.53	023726-93-4	0.06	0.14	0.08
21	Perhydrofarnesyl acetone	1850.32	29.43	000502-69-2	0.09	0.04	/
22	2(1H)-Naphthalenone, 3,4,4a,5,6,7-hexahydro-1,1,4a-trimethyl-	1282.69	18.13	004668-61-5	0.06	0.09	/
Aldehydes							
23	Benzaldehyde	967.64	9.47	000100-52-7	0.18	/	0.09
24	(E,E)-2,4-Nonadienal	994.90	10.36	005910-87-2	0.23	0.19	0.74
25	Benzeneacetaldehyde	1052.32	12.07	000122-78-1	0.18	/	0.36
26	*β*-Cyclocitral	1224.18	16.72	000432-25-7	0.12	/	0.40
27	*β*-Cyclohomocitral	1261.37	17.62	000472-66-2	0.04	0.03	0.10
28	Decanal	1207.83	16.33	000112-31-2	/	0.12	/
29	2-Butyl-2-octenal	2200.49	20.30	013019-16-4	/	/	0.04
Aromatics							
30	1-ethyl-2,3-dimethyl-Benzene	1027.50	11.34	000933-98-2	0.01	/	/
31	Naphthalene	1185.79	15.76	000091-20-3	0.16	0.07	0.29
32	(E)-1,2,3-trimethyl-4-propenyl-Naphthalene	1686.61	26.53	026137-53-1	/	/	0.04
33	1-methyl-Naphthalene	1297.39	18.49	000090-12-0	0.07	/	0.24
34	2-methyl-Naphthalene	2138.02	18.89	000091-57-6	0.02	/	/
35	1,2-dihydro-1,1,6-tri methyl-Naphthalene	2180.66	19.85	030364-38-6	0.02	/	/
36	*α*-Ionene	2183.72	19.92	000475-03-6	0.01	/	/
37	Indole	2134.96	18.82	000120-72-9	0.03	0.08	/
38	Indolizine	2171.03	19.63	000274-40-8	/	0.02	/
Alcohols							
39	Cedrol	1612.75	25.14	000077-53-2	0.10	0.05	0.24
40	Geraniol	1271.76	17.87	000106-24-1	0.12	/	0.88
41	nerol	1276.56	17.99	000106-25-2	0.05	0.08	/
42	*α*-Terpineol	1195.71	16.02	000098-55-5	/	0.16	/
43	Phenylethyl Alcohol	1196.71	14.39	000060-12-8	0.04	/	/
44	Isoborneol	1169.89	15.34	000124-76-5	/	/	0.10
45	Linalool	1102.56	13.55	000078-70-6	1.75	12.40	23.81
46	cis-Linalool oxide (furan)	1075.75	12.76	005989-33-3	0.29	0.09	0.72
47	trans-Linalool oxide (furan)	1090.89	13.21	034995-77-2	0.42	0.44	2.07
48	cis-Linalool oxide (pyran)	1172.48	15.41	014009-71-3	0.29	0.06	0.15
49	trans-Linalool oxide (pyran)	1177.63	15.54	039028-58-5	/	0.17	0.55
Alkanes							
50	Dodecane	1200.70	16.15	000112-40-3	0.26	0.12	/
51	Cyclotetradecane	1449.76	21.85	000295-17-0	0.07	0.04	0.11
52	Cyclopentadecane	2215.17	20.63	000295-48-7	0.05	/	/
53	Tetradecane	1405.18	20.92	000629-59-4	0.20	0.28	0.45
54	Tridecane	2123.56	18.56	000629-50-5	0.09	0.09	0.03
55	Cyclohexadecane	1656.80	25.97	000295-65-8	0.03	0.05	/
56	Hexadecane	1600.27	24.90	000544-76-3	0.16	0.20	0.64
57	Octadecane	1799.94	28.57	000593-45-3	0.01	0.03	0.05
58	Heptadecane	1700.00	26.78	000629-78-7	0.02	0.04	0.07
59	Pristane	1705.75	26.88	001921-70-6	0.01	0.03	/
60	Heptadecane, 3-methyl-	1771.58	28.06	006418-44-6	0.01	0.02	/
61	Pentadecane, 3-methyl-	1571.15	24.33	002882-96-4	0.21	0.16	0.39
62	Dodecane, 2,6,10-trimethyl-	1603.61	24.97	003891-98-3	/	0.03	/
63	Dodecane, 2,6,11-trimethyl-	1462.77	22.13	031295-56-4	0.05	0.04	0.07
64	Phytan	1809.09	28.73	000638-36-8	/	0.02	0.06
65	Eicosane	1500.86	22.93	000112-95-8	0.25	0.29	2.61
66	Octadecane, 2-methyl-	2194.37	20.16	001560-88-9	0.04	0.06	0.08
67	2-cyclohexyl-Octane	1652.87	25.89	002883-05-8	0.01	0.02	/
68	Heneicosane	2099.87	33.46	000629-94-7	0.01	0.01	0.06
69	hexyl-Cyclopentane	1243.35	17.18	004457-00-5	0.03	0.02	/
70	nonyl-Cyclopentane	1449.76	21.85	002882-98-6	/	/	0.11
71	2,6,11,15-tetramethyl-Hexadecane	1706.09	26.89	000504-44-9	/	/	0.05
72	undecyl-Cyclopentane	1656.85	25.97	006785-23-5	/	/	0.05
Esters							
73	Diisobutyl phthalate	1874.55	29.84	000084-69-5	0.07	/	0.46
74	Dibutyl phthalate	1970.46	31.42	000084-74-2	0.03	/	0.05
75	Diethyl Phthalate	1603.61	24.97	000084-66-2	0.04	/	/
76	Phthalic acid, isobutyl octyl este	1872.49	29.81	1000309-04-5	/	0.04	/
77	Hexadecanoic acd, methyl ester	1873.49	30.73	000112-39-0	0.06	0.04	0.08
78	(Z)-3-Hexenyl hexanoate	2205.81	20.42	031501-11-8	0.04	0.17	0.14
79	Dihydroactinidiolide	1542.35	23.75	017092-92-1	0.23	0.07	0.04
80	cis-Geranylacetone	1458.69	22.04	003879-26-3	/	0.09	/
81	Methyl salicylate	1197.63	16.07	000119-36-8	/	/	0.19
82	cis-3-Hexenyl isovalerate	1238.18	17.06	035154-45-1	0.07	0.04	0.18
Phenols							
83	3,5-Di-tert-butylphenol	1516.11	23.23	001138-52-9	0.07	0.06	/
84	2-Methyl-4-hydroxyaniline	1057.16	12.21	002835-99-6	/	0.03	/
85	2,4-Di-tert-butylphenol	1516.16	23.23	000096-76-4	/	/	0.13

Note: No., number; RI, retention index; RT, retention time; CAS, chemical abstracts service; FWT: Fuding white tea; GWT: GABA white tea; YWT: Yunnan white tea.

**Table 2:** Sensory evaluation and score in three types of white tea.

Sample	Shape	Aroma	Color	Taste	Leaf-Base	Total Score
FWT	Color grayish green slightly yellow	Clean-fresh odor	Yellow bright	Fresh and sweet	Green-yellow, symmetrical	94.3
GWT	Buds with leaves, yellow-green color with milli	Milli incense with fruital	Bright yellow	Sweet and rich with a return to sweet	Green-yellow, vein with red	93.4
YWT	Bud with leaves, color gray green show with milli	Fresh and pure	Apricot yellow bright	Sweet and rich	Fat bright, symmetrical	91.5

Note: Total score = shape × 25% + aroma × 25% + color × 10% + taste × 30% + leaf-base × 10%.

**Table 3:** Identification of volatile substances in GABA white tea with anaerobic treatment.

No.	Compounds	RI	RT	CAS	Relative Content	
					GWT	GWT1
1	D-Limonene	1030.78	11.43	005989-27-5	0.15	/
2	trans-*β*-Ocimene	1041.65	11.75	003779-61-1	0.04	/
3	*β*-Ocimene	1051.95	12.06	013877-91-3	0.15	/
4	Caryophyllene oxide	1592.80	24.76	001139-30-6	/	0.03
5	Caryophyllene	1425.55	21.34	000087-44-5	0.17	0.07
6	*α*-Calacorene	1550.15	23.91	021391-99-1	0.04	0.02
7	*β*-Cedrene	1649.84	25.84	000546-28-1	0.03	0.03
8	*δ*-Cadinene	1529.41	23.50	000483-76-1	0.05	0.04
9	*β*-Guaiene	1664.45	26.11	000088-84-6	0.03	/
10	3-Carene	1234.62	16.97	013466-78-9	/	0.18
11	(Z)-3-Tetradecene	2215.22	20.63	041446-67-7	0.05	/
12	Isoshyobunone	1471.18	22.31	1000360-30-1	/	0.03
13	Jasmone	1409.54	21.01	000488-10-8	0.13	0.21
14	*α*-Ionone	1433.71	21.52	000127-41-3	0.07	0.03
15	trans-*β*-Ionone	1491.03	22.72	000079-77-6	0.36	0.21
16	*β*-Damascenone	2210.65	20.53	023726-93-4	0.14	/
17	Perhydrofarnesyl acetone	1850.32	29.43	000502-69-2	0.04	/
18	2(1H)-Naphthalenone, 3,4,4a,5,6,7-hexahydro-1,1,4a-trimethyl-	1282.69	18.13	004668-61-5	0.09	/
19	Benzaldehyde	967.64	9.47	000100-52-7	/	0.00
20	(E,E)-2,4-Nonadienal	994.90	10.36	005910-87-2	0.19	0.21
21	Benzeneacetaldehyde	1052.32	12.07	000122-78-1	/	0.04
22	*β*-Cyclocitral	1224.18	16.72	000432-25-7	/	0.09
23	*β*-Cyclohomocitral	1261.37	17.62	000472-66-2	0.03	/
24	Decanal	1207.83	16.33	000112-31-2	0.12	/
25	2-Butyl-2-octenal	2200.49	20.30	013019-16-4	/	0.05
26	Naphthalene	1185.79	15.76	000091-20-3	0.07	0.04
27	(E)-1,2,3-trimethyl-4-propenyl-Naphthalene	1686.61	26.53	026137-53-1	/	0.02
28	Indole	2134.96	18.82	000120-72-9	0.08	/
29	Indolizine	2171.03	19.63	000274-40-8	0.02	/
30	Cedrol	1612.75	25.14	000077-53-2	0.05	0.25
31	Geraniol	1271.76	17.87	000106-24-1	/	5.76
32	nerol	1276.56	17.99	000106-25-2	0.08	0.36
33	*α*-Terpineol	1195.71	16.02	000098-55-5	0.16	0.09
34	Benzyl alcohol	1059.30	12.28	000100-51-6	/	0.07
35	Phenylethyl Alcohol	1060.30	14.39	000060-12-8	/	0.04
36	Linalool	1102.56	13.55	000078-70-6	12.40	10.70
37	cis-Linalool oxide (furan)	1075.75	12.76	005989-33-3	0.09	0.29
38	trans-Linalool oxide (furan)	1090.89	13.21	034995-77-2	0.44	0.85
39	cis-Linalool oxide (pyran)	1172.48	15.41	014009-71-3	0.06	0.05
40	trans-Linalool oxide (pyran)	1177.63	15.54	039028-58-5	0.17	0.35
41	Dodecane	1200.70	16.15	000112-40-3	0.12	/
42	Pentadecane	1804.40	28.65	000629-62-9	/	0.01
43	Cyclotetradecane	1449.76	21.85	000295-17-0	0.04	0.02
44	Tetradecane	1405.18	20.92	000629-59-4	0.28	0.04
45	Tridecane	2123.56	18.56	000629-50-5	0.09	0.03
46	Cyclohexadecane	1656.80	25.97	000295-65-8	0.05	/
47	Hexadecane	1600.27	24.90	000544-76-3	0.20	0.27
48	Octadecane	1799.94	28.57	000593-45-3	0.03	0.04
49	Heptadecane	1700.00	26.78	000629-78-7	0.04	0.05
50	Pristane	1705.75	26.88	001921-70-6	0.03	/
51	Heptadecane, 3-methyl-	1771.58	28.06	006418-44-6	0.02	0.01
52	Pentadecane, 3-methyl-	1571.15	24.33	002882-96-4	0.16	0.19
53	Dodecane, 2,6,10-trimethyl-	1603.61	24.97	003891-98-3	0.03	/
54	Dodecane, 2,6,11-trimethyl-	1462.77	22.13	031295-56-4	0.04	0.02
55	Phytan	1809.09	28.73	000638-36-8	0.02	0.08
56	Eicosane	1500.86	22.93	000112-95-8	0.29	0.73
57	Octadecane, 2-methyl-	2194.37	20.16	001560-88-9	0.06	0.03
58	2-cyclohexyl-Octane	1652.87	25.89	002883-05-8	0.02	/
59	Heneicosane	2099.87	33.46	000629-94-7	0.01	0.02
60	hexyl-Cyclopentane	1243.35	17.18	004457-00-5	0.02	/
61	nonyl-Cyclopentane	1449.76	21.85	002882-98-6	/	0.04
62	2,6,11,15-tetramethyl-Hexadecane	1706.09	26.89	000504-44-9	/	0.06
63	undecyl-Cyclopentane	1656.85	25.97	006785-23-5	/	0.03
64	Diisobutyl phthalate	1874.55	29.84	000084-69-5	/	0.20
65	Dibutyl phthalate	1970.46	31.42	000084-74-2	/	0.11
66	Phthalic acid, isobutyl octyl este	1872.49	29.81	1000309-04-5	0.04	/
67	Hexadecanoic acd, methyl ester	1873.49	30.73	000112-39-0	0.04	0.11
68	Hexadecanoic acid, ethyl ester	1999.69	31.90	000628-97-7	/	0.07
69	(Z)-3-Hexenyl hexanoate	2205.81	20.42	031501-11-8	0.17	0.02
70	Dihydroactinidiolide	1542.35	23.75	017092-92-1	0.07	0.03
71	cis-Geranylacetone	1458.69	22.04	003879-26-3	0.09	/
72	Methyl salicylate	1197.63	16.07	000119-36-8	/	1.97
73	cis-3-Hexenyl isovalerate	1238.18	17.06	035154-45-1	0.04	0.08
74	3,5-Di-tert-butylphenol	1516.11	23.23	001138-52-9	0.06	/
75	2-Methyl-4-hydroxyaniline	1057.16	12.21	002835-99-6	0.03	/
76	2,4-Di-tert-butylphenol	1516.16	23.23	000096-76-4	/	0.01

Note: No., number; RI, retention index; RT, retention time; CAS, chemical abstracts service. GWT stands for 2023 anaerobically treated white tea; GWT1 stands for 2024 anaerobically treated white tea.

**Table 4:** Volatile substance composition aroma presentation characteristics and classification of GABA white tea with anaerobic treatment.

No.	Compounds	RI	RT	Relative Content		Aroma Description	Category
				GWT	GWT1		
1	Caryophyllene	1425.55	21.34	0.17	0.07	light lilac-like fragrance	A
2	α-Calacorene	1550.15	23.91	0.04	0.02	/	/
3	β-Cedrene	1649.84	25.84	0.03	0.03	/	/
4	δ-Cadinene	1529.41	23.50	0.05	0.04	/	/
5	Jasmone	1409.54	21.01	0.13	0.21	floral, rose like [25]	A
6	α-Ionone	1433.71	21.52	0.07	0.03	warm-woody, balsamic-floral [25] pleasant, cream-like, rose-like [27]	C
7	trans-β-Ionone	1491.03	22.72	0.36	0.21	dry, floral, fruity1 Violet scent [28]	A
8	(E,E)-2,4-Nonadienal	994.90	10.36	0.19	0.21	Strong aromas of flowers, fruits and oils [28]	A
9	Naphthalene	1185.79	15.76	0.07	0.04	non-pleasant, irritating [27]	D
10	Cedrol	1612.75	25.14	0.05	0.25	woody aroma with creamy, very persistent	C
11	nerol	1276.56	17.99	0.08	0.36	sweet scent of fresh roses with lemon	A/B
12	α-Terpineol	1195.71	16.02	0.16	0.09	distinctive lilac aroma	A
13	Linalool	1102.56	13.55	12.40	10.70	sweet, fresh floral aroma	A
14	cis-Linalool oxide (furan)	1075.75	12.76	0.09	0.29	floral [25]	A
15	trans-Linalool oxide (furan)	1090.89	13.21	0.44	0.85	floral [25]	A
16	cis-Linalool oxide (pyran)	1172.48	15.41	0.06	0.05	floral [25]	A
17	trans-Linalool oxide (pyran)	1177.63	15.54	0.17	0.35	floral [25]	A
18	Cyclotetradecane	1449.76	21.85	0.04	0.02		
19	Tetradecane	1405.18	20.92	0.28	0.04		
20	Tridecane	1300.56	18.56	0.09	0.03	hydrocarbo-like [25]	D
21	Hexadecane	1600.27	24.90	0.20	0.27		
22	Octadecane	1799.94	28.57	0.03	0.04	hydrocarbo-like [25]	D
23	Heptadecane	1700.00	26.78	0.04	0.05	hydrocarbo-like [25]	D
24	Heptadecane, 3-methyl-	1771.58	28.06	0.02	0.01		
25	Pentadecane, 3-methyl-	1571.15	24.33	0.16	0.19		
26	Dodecane, 2,6,11-trimethyl-	1462.77	22.13	0.04	0.02		
27	Phytan	1809.09	28.73	0.02	0.08		
28	Eicosane	2000.09	22.93	0.29	0.73	hydrocarbo-like [25]	D
29	Octadecane, 2-methyl-	2194.37	20.16	0.06	0.03	/	/
30	Heneicosane	2099.87	33.46	0.01	0.02	/	/
31	Hexadecanoic acd, methyl ester	1873.49	30.73	0.04	0.11	ester like [25]	D
32	(Z)-3-Hexenyl hexanoate	2205.81	20.42	0.17	0.02	sweet and fruity, apple-pear like	A
33	Dihydroactinidiolide	1542.35	23.75	0.07	0.03		
34	cis-3-Hexenyl isovalerate	1238.18	17.06	0.04	0.08	green, fresh, sweet, floral [26,27]	B

Note: No., number; RI, retention index; RT, retention time; CAS, chemical abstracts service. GWT stands for 2023 anaerobically treated white tea; GWT1 stands for 2024 anaerobically treated white tea. A: fruity; B: fresh; C: woody; D: other.

## Data Availability

The original contributions presented in this study are included in the article. Further inquiries can be directed at the corresponding author.
